# Editorial: Novel insights into sperm function and selection: from basic research to clinical application

**DOI:** 10.3389/fendo.2023.1231545

**Published:** 2023-06-15

**Authors:** Kun Li, Tao Luo, Rossella Cannarella

**Affiliations:** ^1^ School of Pharmacy, Hangzhou Medical College, Hangzhou, China; ^2^ Institute of Life Science, Nanchang University, Nanchang, China; ^3^ Department of Clinical and Experimental Medicine, University of Catania, Catania, Italy; ^4^ Glickman Urology and Kidney Institute, Cleveland Clinic Foundation, Cleveland, OH, United States

**Keywords:** sperm, sperm function, sperm selection, sperm preparation, assisted reproductive technology (ART), male infertility, *in vitro* fertilization, intracytoplasmic sperm injection

Successful reproduction via natural fertilization depends on normal sperm function and selection. The sperm function encompasses motility, capacitation, hyperactivation, acrosome reaction, chemotaxis, thermotaxis, rheotaxis, fertilizing capacity, and so on. All these functions help the sperm to reach the place where it meets, recognizes, and fuses with the egg. Meanwhile, during natural fertilization, malfunctional sperm can be easily recognized in different positions of the female reproductive tract. Namely, sperm selection is also essential for successful reproduction. Along with technological development, selection of the most functional sperm is a key step during *in vitro* fertilization (IVF) and intracytoplasmic sperm injection (ICSI) techniques.

This Research Topic, edited by Kun Li (Hangzhou Medical College, China), Tao Luo (Nanchang University, China), and Rossella Cannarella (University of Catania, Italy, and Glickman Urology and Kidney Institute, USA), focuses on the understanding of the novel trends in sperm function and selection techniques. This Research Topic has collected 13 articles, including 11 original and 2 review articles. The volume has contributed to novel aspects from basic research to clinical application.

## Identifying new gene variants involved in abnormal sperm parameters

Clinical routine testing of semen mainly includes count, motility, and morphology. Motility is included among the most important functional tests. Reduced motility or absent sperm motility, known as asthenozoospermia, often accompanies the abnormal sperm count and/or morphology, collectively referred to as oligoasthenoteratozoospermia or asthenoteratozoospermia. Besides, azoospermia may also be found in semen examination.

New variants in pathogenic genes have been found in infertile men with abnormal sperm parameters. Bai et al. identified four X-linked hemizygous deleterious variants of *TATA-Box Binding Protein Associated Factor 7 Like* (*TAF7L*), including c.1301_1302del (p.V434Afs*5), c.699G>T (p.R233S), c.508delA (p. T170fs), and c.719dupA (p.K240fs). The findings support that *TAF7L* is one of the pathogenic genes of oligoasthenoteratozoospermia. The study by Meng et al. indicates that *Kinesin Family Member 9* (*KIF9*) associates with the central microtubules in human sperm. Bi-allelic *KIF9* loss-of-function variants cause asthenozoospermia. Sha et al. demonstrated for the first time that homozygous *Dynein Axonemal Light Intermediate Chain 1* (*DNALI1*) mutation may impair the integration of axoneme structure, affect sperm motility and cause asthenoteratozoospermia in humans. Yu et al. screened 375 asthenoteratozoospermic patients and identified two novel compound heterozygous variants of *HYDIN Axonemal Central Pair Apparatus Protein* (*HYDIN*), which significantly reduced sperm HYDIN level, damaged flagella structure, and disrupted assembly of the acrosome and neck. Xue et al. summarized the mutations of nine important genes expressed in sperm or oocytes, namely *Phospholipase C Zeta 1* (*PLCZ1*), *Actin-like protein 7A* (*ACTL7A*), *Actin-like protein 9* (*ACTL9*), *dynein axonemal heavy chain 17* (*DNAH17*), *WEE2*, *Tubulin Beta 8 Class VIII* (*TUBB8*), *NLR Family Pyrin Domain Containing 5* (*NLRP5*), *Zona Pellucida Glycoprotein 2* (*ZP2*), and *Transducin-Like Enhancer Protein 6* (*TLE6*), which can contribute to the fertilization failure of IVF/ICSI attempts. These abnormalities mainly showed Mendelian patterns of inheritance, including dominant and recessive inheritance, although *de novo* mutations were present in some cases. Song et al. revealed non-obstructive azoospermia-affected cases with *Testis-expressed gene 11* (*TEX11*) mutations (exon 5, c.313C>T: p.R105*), (exon 7, c.427A>C: p.K143Q) and (exon 29, c.2575G>A: p.G859R), that have not been previously reported. Shi et al. reported thirteen autosomal dominant polycystic kidney disease (ADPKD) males suffering from infertility and investigated the microtubule abnormalities associated with the disruption of *Polycystin-1* (*PKD1*). Their results suggested that the dysregulated Hippo signaling prominently contributed to ciliary anomalies in ADPKD and was potentially associated with axonemal defects in sperm. Liu et al. identified a novel missense mutation (c.1414G>A; p.V472M) in *Cilia and Flagella Associated Protein 47* (*CFAP47*) in two unrelated patients with asthenoteratozoospermia.

In summary, these articles focused on the gene variants involved in abnormal sperm parameters. This research field will give new clues about the etiology of unexplained infertility, which will be beneficial for the diagnosis and treatment of male infertility.

## Discovering new protein biomarkers of sperm function and quality

Potential protein biomarkers were also reported to be linked with the sperm-zona pellucida (ZP)-binding ability and the quality of the frozen sperm. Leung et al. reported that heat shock protein70 2 (HSPA2) and sperm acrosome associated 3 (SPACA 3) are associated with the sperm ZP-binding ability. The results validated the possibility of applying spermatozoa-ZP interaction to select fertilization-competent spermatozoa in assisted reproductive technology (ART). Arunkumar et al. reported that the temperature equilibration process lowered the abundance of sperm proteins in bull, was involved in energy metabolism, structural integrity, and DNA repair, and increased the abundance of proteins associated with proteolysis and protein degradation. The abundance of proteins associated with signal pathways in sperm, such as metabolism, cyclic guanosine 3’, 5’-monophosphate-dependent protein kinase G signaling, and regulation of the actin cytoskeleton.

## Reporting new findings on ICSI at the clinical ART lab

One report showed the effect of different sperm preparation techniques on ICSI’s clinical outcomes. Li et al. evaluated the effect of different sperm preparation techniques on fertilization rate, cleavage rate, embryo quality, endometrial thickness, implantation, biochemical pregnancy, clinical pregnancy, and live birth rates. They found that different sperm sources did not affect the embryo and clinical outcomes after IVM-ICSI cycles, including percutaneous epididymal sperm aspiration, testicular sperm aspiration, and ejaculated sperm.

Another report analyzed the current status and hotspots of ICSI based on 8271 publications between 2002 and 2021. Shen et al. showed that the hotspot topics of ICSI have been risks of ICSI, oocyte preservation, live birth rate, infertile men, and embryo quality in the past two decades. The top five prolific countries have been USA, China, Italy, Japan, and Belgium; the United States accounted for 22.65% of all publications in 2002; after 2018, four countries, China, UK, Italy, and Spain, increased rapidly, and China accounted for 32.10% of all publications on ICSI from 2018 to 2021. The number of publications from China grew exponentially from only five publications in 2002 to 208 publications in 2021; the top five contributing organizations were the Free University of Brussels, University of Copenhagen, University of Valencia, Ghent University, and the University of California San Francisco; the most productive and cited journals were Fertility and Sterility and Human Reproduction. This study presents a research overview of ICSI from different perspectives. These findings will contribute to a better understanding of the current status of ICSI research and provide hotspots and trends for future studies.

## Discussing new developing trends in methods for sperm selection or sperm function evaluation

Nixon et al. reviewed recent developments in the understanding of sperm biology and function and highlighted the development of cutting-edge approaches for identifying and treating male infertility. In particular, the review focused on the progress toward the implementation of precision medicine and the application of advanced technology platforms, including whole exome sequencing, proteomic analyses, advanced imaging technologies, and machine learning artificial intelligence. The review showed that the increasing novel mechanistic understanding of sperm biology and function, and the improvement of advanced technology will have a deep impact on many aspects: the uncovered and expanding potential candidate biomarkers, diagnostics, and treatments of male infertility, disrupting the fertility care paradigm, optimizing outcomes for the management of male infertility.

In conclusion, research published in this Research Topic revealed new gene variants and proteins correlated with sperm function and quality, new findings of sperm preparation, the research trends and hotspots on ICSI at clinical ART labs, and new developing trends in methods for sperm selection or function evaluation. This Research Topic has increased the insights from basic research of the genes and proteins that may affect sperm function and sperm selection to clinical application, which is beneficial for the fields of reproductive biology and reproductive medicine.

## Author contributions

KL, TL, and RC drafted and revised the manuscript. All authors have read and confirmed this editorial for publication.

